# Supplementary material for: Chromothripsis during telomere crisis is independent of NHEJ, and consistent with a replicative origin
Source: Genome Res. 2019 May;29(5):737–49. doi: 10.1101/gr.240705.118 (PMC6499312; doi:10.1101/gr.240705.118)
Supplement: Supplemental Material [file supp_gr.240705.118_Supplemental_file_1.zip › contigs/annotated_contigs/DB84/contig.2.DB84_length_655_mean_cov_9.16030534351.docx]

**DB84_length_655_mean_cov_9.16030534351**

```
ACCTTTTTATTTACAAAAAGAAGTTGTTTCTAACATATTCTTCTTACAAGTGTTACTTTTCCATAAATATAGGATAGAGGAAATGAAAT
 >chrX:146734066-146734390 + E=3e-183
GCACAGCGACATATCTACCTTTATACACTGTAGAAAACACTATAGAACGTTTAGAACTAGCAAAGGAATAGTTCTATCTCATTTGTTAT

TTTTATAGTCTAGTGATTTTGTGGAATCACTACATCATGGTGTAAATGATGTATAAAATGAAAAATGATCAGTTTCCTTTGCATGGAGA

ATATAGAGGATGCTTTGTAATTTATTTCCTACCTGTACTGTTGGCTACAGTAAA|AGC|AGCAGCTCGTTTGTTTGTAATTAGAATTCT
                                                       >chrX:146761457-146761791 + E=3e
GAATTTTCGTAAGATTTGTGTAGGATTCATGATCTTAAATGCTTTAGAACCACCATCATAAAATAAATTCAATTTTTCTGAAGGTAGGC
-186
ATTTTAATTGCCTGCTCTTCACCTCATTGATGAGGGAGGGATTCACAGCCTAGGGTTATGGAGATGGCCAAACATGACACCTGACACTG

CACAGGTGAGATCAATAGCAATTTGTCAGTCACAATCCAGTGGAGGAAGACACTACACACCATGCAGGGCCAAACAGGGGTTGCACTTG

TAAAGAGTGAACATGTAATCACTGTGGGAGGCAG
```
